# Histamine as an emergent indoor contaminant: Accumulation and persistence in bed bug infested homes

**DOI:** 10.1371/journal.pone.0192462

**Published:** 2018-02-12

**Authors:** Zachary C. DeVries, Richard G. Santangelo, Alexis M. Barbarin, Coby Schal

**Affiliations:** 1 Department of Entomology and Plant Pathology, North Carolina State University, Raleigh, North Carolina, United States of America; 2 W.M. Keck Center for Behavioral Biology, North Carolina State University, Raleigh, North Carolina, United States of America; 3 NC Department of Health and Human Services, Communicable Disease Branch, Raleigh, North Carolina, United States of America; 4 Center for Human Health and the Environment, North Carolina State University, Raleigh, North Carolina, United States of America; University of Cincinnati, UNITED STATES

## Abstract

Histamine is used in bronchial and dermal provocation, but it is rarely considered an environmental risk factor in allergic disease. Because bed bugs defecate large amounts of histamine as a component of their aggregation pheromone, we sought to determine if histamine accumulates in household dust in bed bug infested homes, and the effects of bed bug eradication with spatial heat on histamine levels in dust. We collected dust in homes and analyzed for histamine before, and up to three months after bed bug eradication. Histamine levels in bed bug infested homes were remarkably high (mean = 54.6±18.9 μg/100 mg of sieved household dust) and significantly higher than in control homes not infested with bed bugs (mean < 2.5±1.9 μg/100 mg of sieved household dust). Heat treatments that eradicated the bed bug infestations failed to reduce histamine levels, even three months after treatment. We report a clear association between histamine levels in household dust and bed bug infestations. The high concentrations, persistence, and proximity to humans during sleep suggest that bed bug-produced histamine may represent an emergent contaminant and pose a serious health risk in the indoor environment.

## Introduction

**Fig 1:**
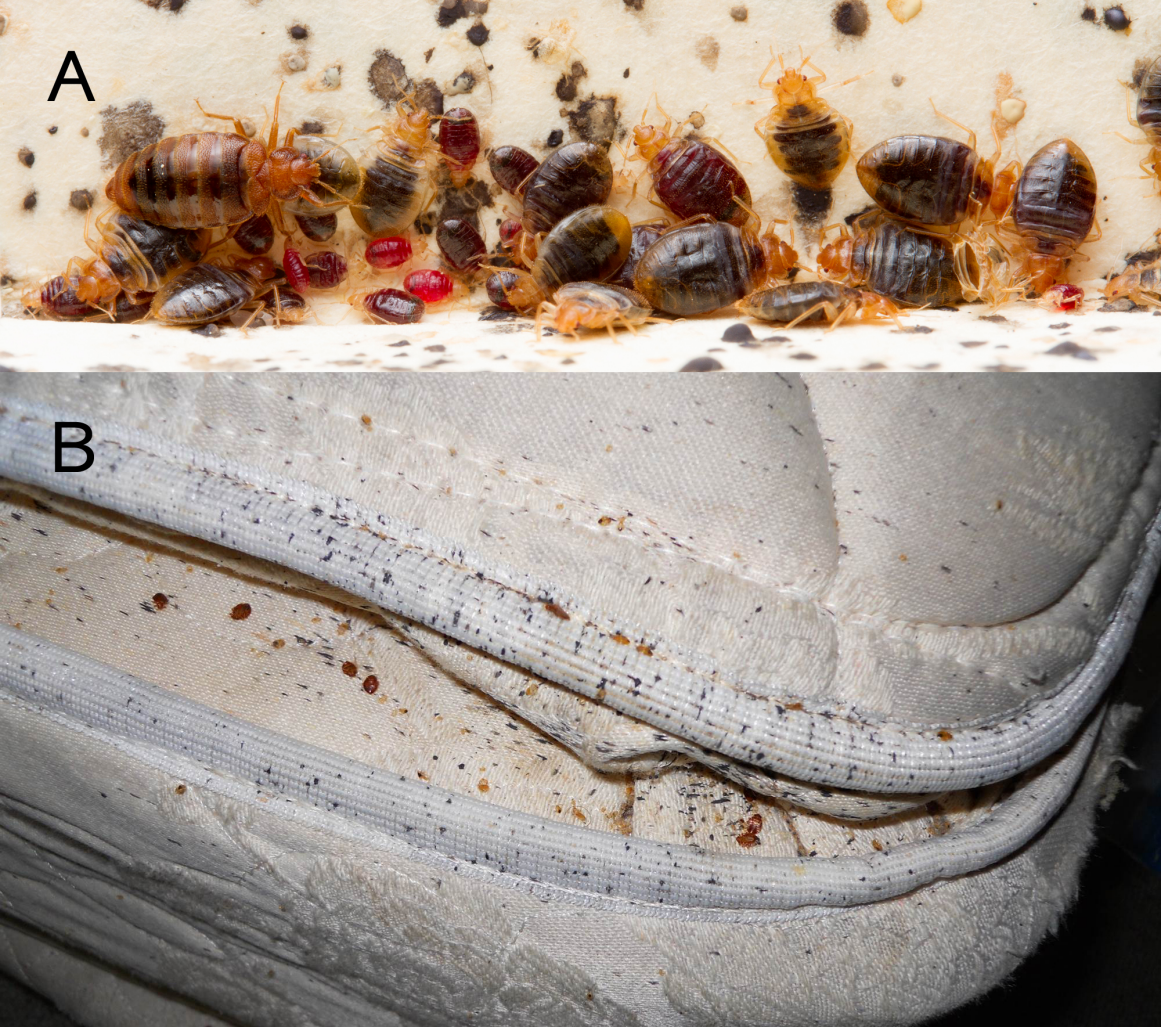
Bed bugs and signs of an infestation. Photos depiciting (**A**) a typical bed bug aggregation showing blood-fed and unfed bed bugs and fecal spots that contain histamine (photo credit: Matt Bertone), and (**B**) a matress heavily stained by bed bug feces, which contains histamine (photo credit: Mike Waldvogel and Jung W. Kim).

Indoor environmental contaminants pose serious health risks to humans. Well-investigated examples include human-generated contaminants, such as lead [1], asbestos [2] and various pesticides [3, 4], along with aeroallergens produced by pests such as house dust mites [5], cockroaches [6], and rodents [7]. These pests share several common features: (a) they are often obligatorily associated with humans and present in large numbers; (b) some of the allergens they produce are excreted in feces and urine and persist in household dust; (c) environmental conditions (sanitation, temperature, humidity) can influence both pest populations and the persistence of allergens; and (d) allergen-containing household dust can become airborne and inhaled when disturbed [8–11]. Bed bugs (*Cimex lectularius*, Fig 1) share these features, but they are not known to produce allergens beyond those delivered with their bites.

Bed bugs had all but disappeared from developed areas of the world by the 1960s, but have resurged globally since the early 2000s [12–17]. Although *C*. *lectularius* is capable of transmitting *Trypanosoma cruzi* (the causative agent of Chagas disease) [18] and *Bartonella quintana* (trench fever) [19] in laboratory assays, there is no evidence of natural pathogen transmission. Bed bug bites, however, can occasionally cause severe dermatitis [20], as humans become sensitized to nitrophorin proteins in bed bug saliva [21]. Bed bugs also have strong psychological impacts on humans, with effects often lasting past their eradication [22]. Bed bug eradication is particularly challenging because of high levels of resistance to insecticides, difficulty applying insecticides onto and close to beds, and costs associated with pest abatement [23]. High temperatures (“heat treatments”) can eliminate bed bugs, but this approach is often prohibitively expensive.

Histamine was recently identified as a close-range aggregation pheromone component in bed bug feces [24], with bed bugs likely synthesizing a majority of histamine *de novo* [25]. Histamine is a known food contaminant, mainly of fish and alcoholic beverages such as beer and wine [26]. When ingested, histamine is a mediator of acute inflammatory reactions and associated with significant adverse effects in sensitive individuals, including hypotension, uticaria, shock, heart palpitations, diarrhea, vomiting, pain, itching, and respiratory distress [26]. Cutaneous exposure is known to result in dermatitis, primarily in atopic patients [27, 28]. Respiratory exposure to histamine can reduce forced expiratory volume (FEV) [29], and increase nasal mucosa reactivity [30], with these effects mostly seen in atopic individuals [31]. Dermal, nasal, or respiratory responses (e.g., bronchial reactivity) [32, 33] to histamine in clinical tests suggest that exposure to histamine in the environment would constitute a significant health risk, although information on environmental exposure is limited. Histamine levels have been correlated with the mass of respirable agricultural dust, and thought to be associated with occupational asthma, rhinitis, bronchitis, and related respiratory syndromes [34–36]. However, information on indoor histamine exposure is scarce, and the health risks from chronic respiratory exposure to histamine are poorly understood.

To determine whether bed bugs deposit histamine and whether histamine could pose an exposure risk for humans indoors, we designed a study to quantify histamine levels in homes with and without bed bugs. In addition, we evaluated the persistence of histamine following bed bug eradication.

## Methods

### Ethics statement

The North Carolina State University Institutional Review Board (IRB) approved this study (#3840). Before participation, adult participants (>21 yrs old) provided informed consent.

### Study design and sampling

Apartments were located within the same nine-story multi-unit (140 apartments) building in Raleigh, NC. This building has been chronically infested with bed bugs for several years despite recurrent pest control interventions. Therefore, some “un-infested” apartments in this building could have been infested with bed bugs prior to our sampling, or they might represent marginally infested units where we failed to detect low-level bed bug populations. To obtain truly un-infested homes, five additional homes in Raleigh, NC, not associated with the apartment building, were also sampled as external negative controls. Unlike the un-infested units in the apartment building, these homes had no evidence of bed bugs in >3 years, and were located >8 km away from the apartment building.

Residences were surveyed and divided into groups based on their infestation status: infested (14 apartments), un-infested within the same complex (10 apartments), and un-infested >8 km away (5 apartments). The bed bug infested homes were further divided into two treatment groups: infested-controls (no intervention; 9 apartments) and infested-treated (intervention; 6 apartments, some switched over from the infested control group). House dust was sampled in all homes at the beginning of the study (baseline), and infested-treated homes were also sampled repeatedly at 2, 4, and 12 weeks after the intervention. Since all homes remained occupied throughout the study, ethical considerations dictated that infested-control homes could be sampled only up to 4 weeks after baseline, and some infested-control apartments subsequently were switched to the infested-treated arm. However, sampling from control and treated apartments overlapped temporally, with staggered start dates.

Homes were visually inspected for bed bugs during the initial home visit. This was followed by sampling bed bugs with traps (Climbup Interceptor, Susan McKnight Inc., Memphis, TN) for two weeks. Climbup Interceptors have previously been shown to be effective at detecting even low-level bed bug infestations [37]. Although quantitative in nature, the trap counts were intended to indicate only the presence or absence of bed bugs, direct our dust sampling efforts, and assess treatment efficacy. Dust samples were collected from an area with the highest concentration of bed bugs in either the bedroom or living room. An area of the floor (3 m x 15 cm) near a wall and behind a bed or couch was sampled for 2 min using a Eureka Mighty-Mite 9.0-ampere vacuum cleaner (Eureka Company, Bloomington, Ill) fitted with a Dustream® collector and filter (40 μm, Indoor Biotechnologies Inc., Charlottesville, VA). Samples were placed into glass vials and stored at -80°C.

### Bed bug interventions

A professional pest control company was contracted by the building management to handle all bed bug abatement efforts. This was entirely independent of our research; our only contact with the pest control company was to coordinate our sampling efforts with their interventions. Bed bug interventions included heat treatments, where the ambient temperature was raised to ~50°C and maintained for >4 hr while fans circulated air throughout the apartment [23]. Following heat treatments, the pest control technician applied residual insecticide sprays and dusts to bed bug sheltering areas. Although we did not participate in the intervention efforts, we actively monitored bed bugs throughout the study using visual inspections and traps. Homes where bed bugs were detected at any time post-intervention were discontinued in the infested-treated arm of the study, and only used for their baseline histamine values.

### Histamine analysis

Dust samples were weighed (total mass), sieved (450 μm), and weighed again (sieved mass). Sieves were made from adhering 450 μm mesh (Wildco®, Yulee, FL) to hollowed out plastic Petri dish lids and bases (4.5 cm diameter; Falcon®, Brookings, SD). Approximately 5–50 mg sieved dust was extracted in plastic centrifuge tubes (Sarstedt Inc., Nümbrecht, Germany) in 1 ml of HPLC grade water. To this mixture, 10 μg of histamine-α,α,β,β-d_4_ (Sigma-Aldrich Co. LLC, St. Louis, MO) was added (in 0.1 M HCl) as an internal standard. Samples were shaken on a rocker for 10 min, centrifuged at 2000 rpm, and the supernatants were transferred to glass vials. To each supernatant the following were added: 1 ml toluene, 2 ml alkaline buffer solution (pH = 12; Fisher Scientific, Hampton, NH), and 100 μl of the derivatization agent isobutyl chloroformate (IBCF; Sigma-Alrich Co. LLC). Samples were then capped and shaken on a rocker for 45 min. Derivatized samples were centrifuged briefly, and the top (organic) layer was transferred to a new glass vial. Samples were blown to dryness under a gentle stream of high-purity nitrogen and heat (30°C), resuspended in 1 ml of toluene, and stored at 4°C.

Samples were analyzed using an Agilent Technologies (Santa Clara, CA) 6890N GC coupled to an Agilent Technologies 5975 mass spectrometer (GC-MS) and operated in EI mode. The GC was fitted with a 30 m x 0.25 mm x 0.25 μm (5%-phenyl)-methylpolysiloxane Agilent J&W HP-5ms column preceded by a 3 m deactivated guard column. The temperature program was: 100°C to 320°C at a rate of 10°C/min, and held at 320°C for 10 min. The IS method of quantification was used with a 9-point calibration curve ranging from 0.1 μg/ml to 50 μg/ml, and no samples exceeded the upper level of the calibration curve. Quantification ions were selected for both the IS (197) and histamine (194), and both compounds eluted at approximately the same time (14.3 min).

### Statistical analysis

The mass values of sieved dust at baseline were compared among the three groups–infested, un-infested in the same building, and un-infested >8 km away–using a one-way ANOVA. Baseline histamine values (before intervention) were cube-root transformed to assure normality, and compared among the infested and un-infested treatment groups using a one-way ANOVA. Changes in histamine over time in either the infested-control homes or infested-treated (intervention) homes were normalized (percentage of baseline value) and evaluated using repeated measures ANOVA with square-root transformed percentages compared using the Tukey-Kramer multiple comparison test. All statistical analyses were performed using SAS 9.4 (SAS Institute, Cary, NC), and all relevant data are available in the supporting information files for this manuscript.

## Results

### Baseline histamine levels in homes

**Fig 2:**
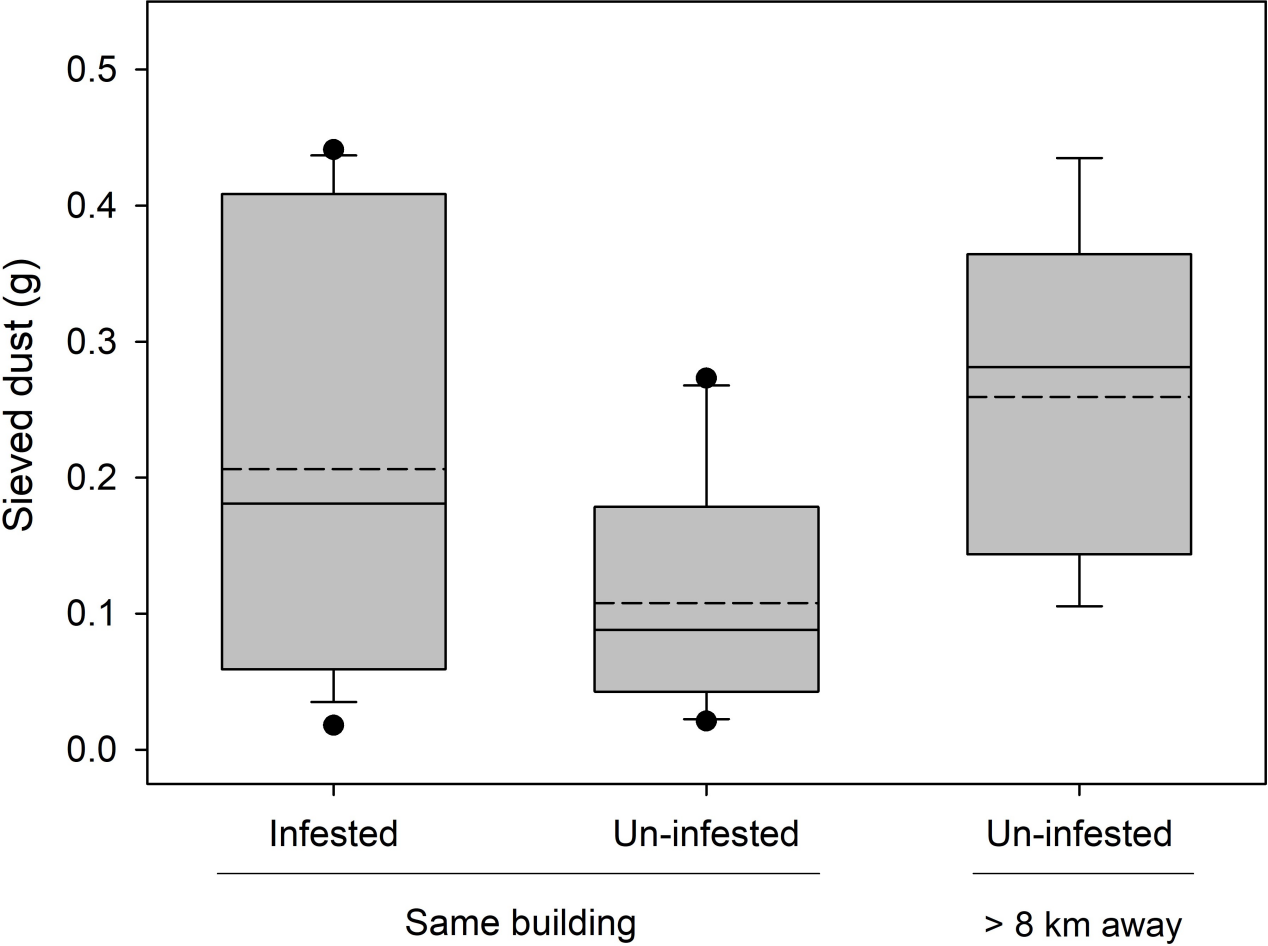
Sieved dust collected from homes. Sieved dust (box plot; mean = dotted line, median = solid line) from house dust collected from bed bug-infested homes (n = 14) and un-infested homes (n = 10) in the same apartment building. Un-infested-control homes (n = 5) are separate apartments >8 km from the apartment building and are not known to have had bed bugs in the past 3 yrs. No significant differences were detected among groups according to ANOVA.

**Fig 3:**
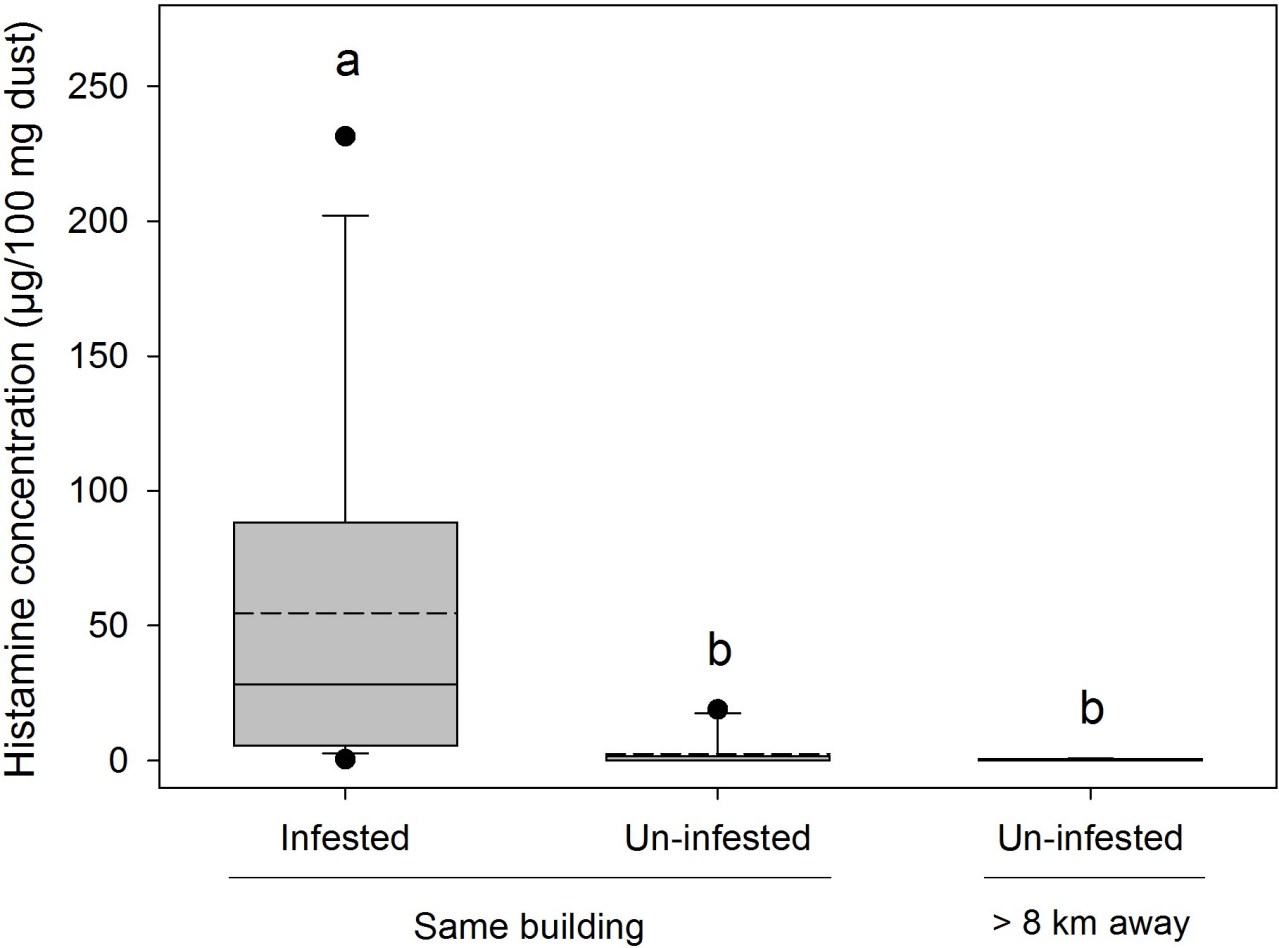
Histamine in bed bug infested homes. Histamine concentrations (box plot; mean = dotted line, median = solid line) in house dust collected from bed bug-infested homes (n = 14) and un-infested homes (n = 10) in the same apartment building. Un-infested-control homes (n = 5) are separate apartments >8 km from the apartment building and are not known to have had bed bugs in the past 3 yrs. Significant differences according to the Tukey-Kramer multiple comparison test (on cube-root transformed data) are indicated by different letters.

In infested homes at baseline, we trapped and visually observed bed bug levels ranging from 16–100 (mean = 40.6 ± 6.2 bed bugs). There were no significant differences in the amount of sieved dust recovered among the three treatment groups (F_2,26_ = 2.72, P = 0.0848, Fig 2). Household dust from homes with active bed bug infestations had significantly higher histamine levels than dust from un-infested-control homes either from the same apartment building or from homes >8 km away (F_2,26_ = 14.84, P < 0.0001, Fig 3). Bed bug-infested homes averaged >54 μg histamine/100 mg dust, while control un-infested homes averaged <2.5 μg/100 mg of dust (same apartment building) or <0.3 μg/100 mg of dust (different buildings, >8 km away).

### Effects of spatial heat treatments on histamine levels

**Fig 4:**
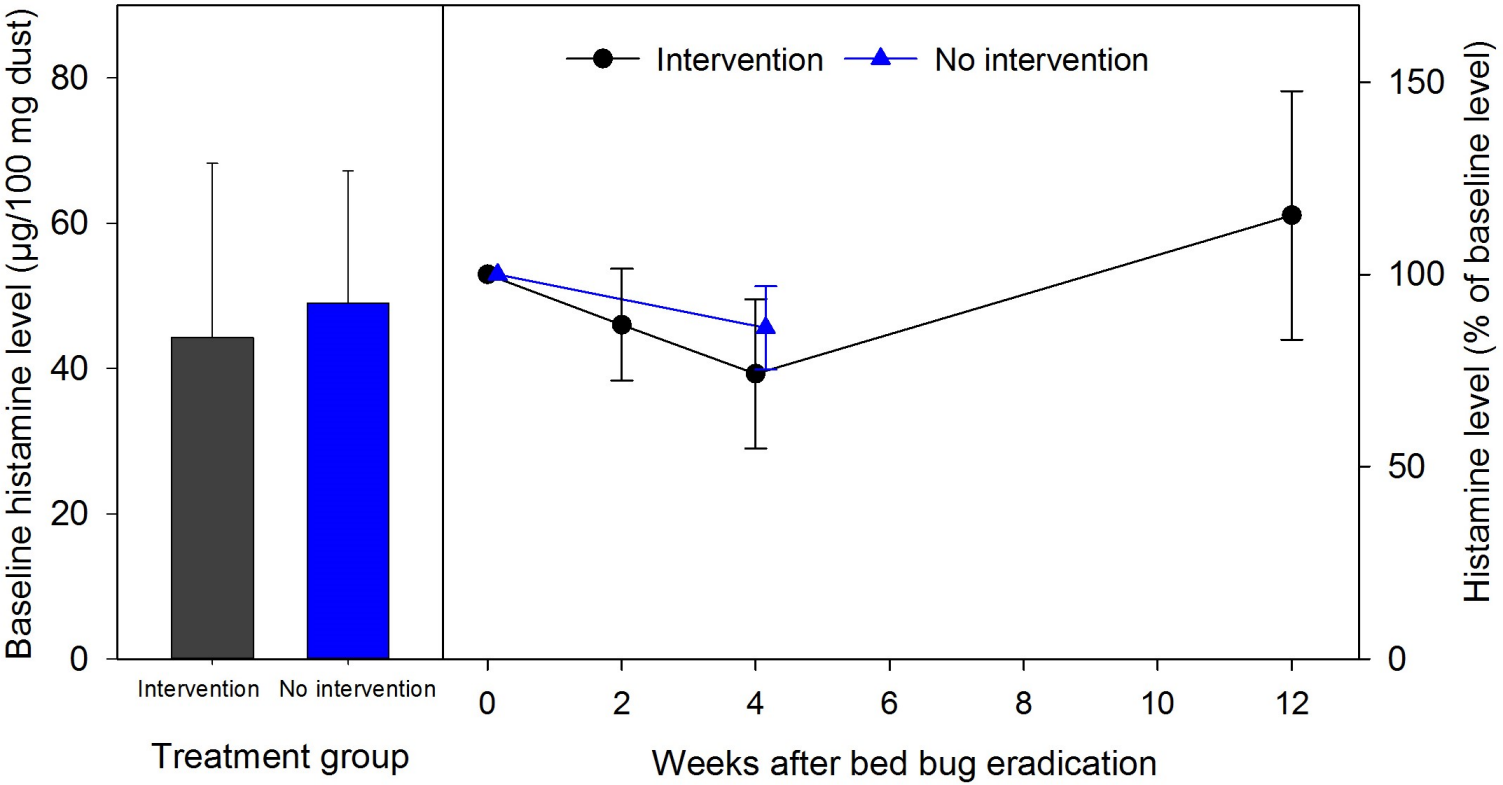
Change in indoor environmental histamine following bed bug eradication. Percent changes in histamine concentrations (mean percentage ± SEM) in house dust collected from bed bug infested control homes (no intervention, n = 9), and bed bug infested treated homes (intervention at time 0, n = 6). No significant differences were observed for either treatment over time.

Histamine levels remained unchanged over one month in no-intervention infested apartments (F_1,8_ = 2.07, P = 0.1878, Fig 4). Heat treatments/interventions (and apparent bed bug eradication) did not significantly affect histamine levels in infested homes over time (F_3,13_ = 0.90, P = 0.4662, Fig 4). It should be noted that one infested home that did not have measurable amounts of histamine at baseline was not used for repeated measures analysis, but was included in the above baseline data. This indicates that (a) histamine in household dust was not degraded by the heat treatment, and (b) histamine is highly stable in household dust and persists for months following bed bug eradication.

## Discussion

Histamine was detected only at trace levels in homes known to be free of bed bugs, indicating that environmental histamine is of no concern in residences that are truly un-infested. On the other hand, we found a clear association between high levels of histamine in house dust and the presence of bed bugs, indicating that bed bugs are the major contributor to indoor histamine residues. Low, but detectable, levels of histamine in some un-infested apartments within the same building suggest either that bed bugs had been present in these apartments at some prior time, or that some bed bugs were present but we failed to detect them.

The significance of these finding is substantial, because exogenous histamine can provoke allergic responses and asthma. Histamine receptors are expressed in many cell types, including epithelial and endothelial cells, dendritic cells, and neutrophils, and in various tissues, including lungs, skin, gut, the nervous system, and the immune system. The histamine concentrations we measured in sieved household dust (mean >54 μg/100 mg dust) are 10-fold higher than the US-FDA upper limit in edible fish (5 mg/100 g fish) [38]. Arguably, comparisons of ingested and inhaled histamine are challenging because airborne limits have not been defined. In clinical diagnostic tests of airway hyper-responsiveness in patients with airway disease, however, patients who breathed saline aerosols containing as little as 24.5 μg of histamine experienced a 20% reduction in forced expiratory volume in one second (FEV_1_) [39]. Additionally, histamine is a potent inducer of pruritus, and cutaneous applications of histamine induce atopic dermatitis [27]. Histamine is used as a positive control in skin-prick allergy tests at a dose of ~0.5 mg (1 drop of a 1% solution) [33]. Unfortunately, there is no information on the health effects of chronic low-level exposure to histamine because prior to our study there was no compelling need for such an assessment.

Quantifications of environmental histamine are scarce. Histamine was detected in dairy farm hay dust at up to 0.7 μg/100 mg of airborne dust and up to 0.05 μg/100 mg of bulk hay [36]. While these levels were far lower than those used in clinical bronchial provocation, they were nevertheless considered a health concern. Notably, the histamine concentrations in dust collected in bed bug-infested homes were 50-times greater than in agricultural hay.

Importantly, the high concentrations of histamine we recovered were from sieved dust particles which readily become airborne and represent the major route of entry of allergens into the airway, as documented in studies correlating cockroach allergens in settled and airborne dust [40]. The potential health risks associated with bed bug-produced histamine might rival those associated with other indoor pests, namely cockroaches and dust mites. Although unknown, we speculate that environmental histamine may have additive or synergistic interactions with other allergens [41]. Because histamine is downstream in the mammalian immune response, exposure to dust-associated airborne histamine would likely trigger more rapid adverse effects than exposure to aeroallergens alone. Histamine produced by bed bugs could potentially pose a greater risk than some arthropod-produced allergens, due to its deposition location and stability. Cockroaches deposit allergens mainly in the kitchen [42, 43]. In contrast, bed bugs, like dust mites [44], aggregate and defecate near their human host (Fig 1), and histamine is deposited on or near where humans sleep and spend arguably the longest amount of time during the day. Therefore, like potent dust mite allergens, bed bug-derived histamine may pose health risks due to its proximity and persistence in our breathing space. In addition, cutaneous exposure to histamine that has accumulated on bedding materials may be of concern. Histamine appears to induce developmental effects, including suppression of epidermal differentiation and thinning of the epidermis, which impairs the barrier function of the skin [45, 46]. Persistent contact with high levels of histamine may therefore pose significant dermal risks.

All bed bug interventions, including chemical and heat treatments [23], singularly focus on bed bug abatement because the objectives are to reduce or eliminate bites. Our finding of histamine persistence at least three months after bed bug eradication underscores the need to develop and validate intervention strategies that eliminate histamine, in addition to eradicating bed bugs. Prolonged exposure of homes to temperatures of 50°C did not reduce histamine levels in house dust. Therefore, a combination of deep cleaning and pest elimination will likely be needed, similar to the strategies used to reduce German cockroach allergens [43]. We suggest that a similar protocol be developed for bed bugs, and evaluated for its efficacy in reducing histamine levels in homes, and ultimately reducing adverse health effects. Additionally, when developing abatement strategies, we must also consider their effects on the movement and distribution of histamine in homes. It is plausible, for instance, that the high temperatures and air circulation during heat treatments could re-circulate histamine-containing dust particles and deposit them in new locations with higher chances to become contacted, inhaled, or consumed.

## Conclusions

Bed bugs have become a major social, economic, and health problem since their global resurgence in the early 2000s. Infestations can reach exceedingly high levels, especially among the elderly and in disadvantaged communities, where interventions may be unaffordable. While bed bug bites have been recognized as a dermatological concern that can be exacerbated and lead to secondary infections, bed bugs have not been implicated as disease vectors or allergen producers. The results of this study demonstrate that the presence of bed bugs strongly correlates with histamine levels in homes, and thus may adversely affect the health of residents through exposure to exogenous histamine. Furthermore, bed bug eradication with heat and insecticides does not appear to reduce histamine levels in homes, suggesting high thermal and chemical stability of histamine. Future investigations should first expand the sample size of the present work, to ensure that our findings are not confounded by any other undetected variables. In addition, future studies should evaluate temporal and spatial dynamics of histamine deposition in bed bug-infested homes, health impacts of dermal and respiratory exposure to environmental histamine, and the efficacy of various mitigation strategies to reduce histamine in homes. The intimate association of bed bugs with humans and the spatial distribution and persistence of histamine in homes suggest that histamine may represent an emergent indoor environmental contaminant whose impact on human health should be investigated.

## Supporting information

S1 Dataset Histamine in household dust dataset. (XLSX)
